# The role of social inequalities in the epidemiology of urological cancers: can this inform cancer screening?

**DOI:** 10.1308/rcsann.2023.0096

**Published:** 2024-05-15

**Authors:** MG Cumberbatch

**Affiliations:** University of Sheffield, UK

**Keywords:** Cancers, Hunterian, Inequalities‌, Lecture, Role, Social

## Abstract

Health inequalities are systematic and potentially remediable differences in health across populations. Understanding the origins of these discrepancies, the healthcare consequences and the manifestations of related diseases can help improve the outcomes of underserved communities. Here I discuss how social factors may be used to help identify particular at-risk populations with regards to urological malignancies, and how these can be potentially used as biomarkers that inform cancer screening targets.

## Commentary

The notion of poverty in the United Kingdom (UK) was perhaps popularised in the late 1800s by the philanthropists Charles Booth and Seebohm Rowntree in their studies of quality-of-life disparities in London and York. Since then, research on the gap between low- and high-income families has developed.^1,2^ Over the past 30 years there has been growing epidemiological evidence of a difference in health outcomes between people from different groups in society.^3^ Health inequalities are systematic and potentially remediable differences in health across populations. Often these differences related to socioeconomic status.^3^ In 2006, the National Health Service made health inequalities one of its top six priorities and introduced the Health Inequalities National Support Team. The Marmot Review in 2010 revealed that although overall life-expectancy has been increasing, the gap between top and bottom has persisted and occasionally widened (updated 2017).^3^ In the 1970s, Julian Tudor-Hart described the inverse care law that described a situation in which those who need care the most, receive the least.^4^ Such a conclusion has also been seen within the Black Report 1980, the Acheson Report 1998 and the Dilnot Report.^5^^–7^

Bladder cancer and kidney cancer are the 11th and 7th most common cancers in the UK, respectively, with 10,300 new bladder cancers and 13,300 new kidney cancers per year. Both cancers have a male preponderance, but bladder cancer in females has a higher mortality rate. Bladder cancer incidence in female patients is 40% greater in deprived areas.^8^ Since the 1990s the incidence of kidney cancer has increased 88%. Around 1,000 kidney cancer cases per year are linked to deprivation.^9^

Bladder cancer is a common malignancy and is one of the most expensive to treat.^10^ The incidence of bladder cancer increases with tobacco smoking and chemical exposure. These factors lead to geographical distributions in incidence and mortality that mirror risk factors and resources. Between 1996 and 2010, the deprivation gradient in incidence rates between the most- and the least-deprived groups stayed the same for most cancer types, including bladder cancer.^11^

In other cancers it has been shown that targeted screening of at-risk, deprived populations confers a survival benefit. For example, currently in the UK there is a screening trial for lung cancer, which entails community-based chest computed tomography scanning for current or previous smokers aged 55–80 years. This follows work in the United States (US), which has shown a survival benefit from screening hard-to-reach at-risk populations near their home.^12,13^ This has been encouraging because bladder cancer and lung cancer share risk factors and commonly occur in the same groups (older males) who often present late to healthcare professionals.^14^ Likewise, in 2009, Greenlee *et al* published data suggesting that county-level poverty in the US was an independent predictor of late-stage at diagnosis for bladder cancer.^15^ Furthermore, the 2010 National Cancer Patient Experience Survey in England showed that both females and patients from minority ethnic groups have a higher probability of three or more prereferral consultations for suspected cancer compared with their white male counterparts. This may have implications for the stage of disease at diagnosis on onwards care options and outcomes.^16^

To fully understand this burden of disease, we began by updating the literature on the epidemiology of these two cancers, primarily through systematic reviews and meta-analyses.^17,18^ What I observed and confirmed, was the higher incidence of bladder cancer in workers performing ‘blue-collar’ tasks within industrial occupations that are associated with less-affluent socioeconomic groups. Furthermore, we were able to show in the largest to date meta-analysis, that ‘current’ smoking status conferred the greatest risks with regards bladder cancer diagnosis.^19^ Data from the Office of National Statistics have illustrated that deprived persons are four times more likely to smoke tobacco than those living in more-affluent areas.^20^

More recently, we examined the differential mortality rates between patients living in greater poverty. The Index of Multiple Deprivation (IMD), a nine-point scale commonly used to measure relative social deprivation, is derived from local rates of income, employment, education, crime and housing, using them to weight the score accordingly. The IMD is allocated using English postcodes and groups people into quintiles, with IMD category 1 being the most socially deprived. For bladder cancer, there was an 8.9% survival difference at 3 years between IMD 1 and 5 in males and a 13.4% difference in women.^21^ A similar but less stark difference was also seen in kidney cancer.

Building on this platform, we developed the concept for the YORKSURe Trial (ISRCTN34273159) funded through Yorkshire Cancer Research, acquiring £1.5million to deliver the feasibility study. This trial consists of three cohorts, examining the feasibility of national community-based screening for bladder cancer (but will detect some early kidney cancer). Early patient and public involvement work had shown that barriers to early diagnosis in those with bladder cancer (and kidney cancer) who are from more-deprived neighbourhoods were the need to take time off work for appointments and the travel time taken to access healthcare, hence identifying an unmet need. This earlier work was funded through an awarded Action Bladder Cancer grant. At the heart of the YORKSURe Trial is the concept of ‘levelling-up’ access to diagnostic tests by placing it in the community (starting in the home using self-test kits interpreted by smartphones), by identifying those at risk via electronic health records (based on age, smoking and sex), and engaging those populations early in the pathway, with the hope of stage-migrating those at greatest risk and improving both morbidity (less-invasive care needed) and mortality outcomes (cancer detected at localised stages). This year, we are building a similar approach to community-based prostate cancer screening, a disease that has shown disparities in stage at diagnosis and also treatment decisions among underserved populations, in particular, differences between ethnic minority groups.

One of the difficulties found when approaching this research is that the effects of social deprivation on health are multifactorial. They can be linked to healthcare access, smoking history and biological cancer behaviours in different populations. A single measure of deprivation is unlikely to be precise enough to elucidate such nuances. However, improving consciousness in the scientific community about the health needs of underserved populations remains of tantamount importance, and social biomarkers are likely to play a key role in cancer screening.
